# Community Participation Approaches for Effective National COVID-19 Pandemic Preparedness and Response: An Experience From Oman

**DOI:** 10.3389/fpubh.2020.616763

**Published:** 2021-01-26

**Authors:** Huda Al Siyabi, Said Al Mukhaini, Mohsen Kanaan, Sumaya Al Hatmi, Zahir Al Anqoudi, Ahmed Al Kalbani, Zakiya Al Bahri, Chadia Wannous, Salah T. Al Awaidy

**Affiliations:** ^1^Directorate General of Primary Health Care, Ministry of Health, Muscat, Oman; ^2^Directorate General of Health Services, Ministry of Health, Al Buraimi Governorate, Oman; ^3^Directorate General of Health Services, Ministry of Health, Ad Dhakhilya Governorate, Oman; ^4^Directorate General of Health Services, Ministry of Health, Muscat Governorate, Oman; ^5^Future Earth-Health Knowledge, Action Network & Toward A Safer World Network (TASW), Stockholm, Sweden; ^6^Office of Health Affairs, Ministry of Health, Muscat, Oman

**Keywords:** COVID-19, community participation, healthy cities, healthy villages, Willayat health committees, volunteers, Oman

## Abstract

Oman, like other countries in the world, was affected by the COVID-19 pandemic. Since the WHO's declaration of the pandemic, the Ministry of Health of Oman has initiated its preparedness and response to the pandemic, with community participation as one of the key components of the national preparedness and response plan. This paper is a descriptive study aims at describing the three community approaches that exist in Oman and reviewing their role in preparedness and response strategies to COVID-19 pandemic and discuss the lessons learned. Community participation approaches in Oman were translated into action during the pandemic through empowering community members, mobilizing resources, and strengthening the ownership among the local community to ensure effective advocacy, proper networking, and dissemination of information and, subsequently, actions at the level of the community. The first community participation approach is community organizations within the healthy cities and villages initiative, which facilitated networking and acted as a platform for community engagement, reviewing the health information and updating them accordingly to meet evloving demands. The second approach is Willayat (District) health committees, with their unique multi-sectoral structure, that enhanced collaboration at the state level with different community leaders and groups to develop pandemic action plans, which were implemented using available local resources. The third approach is community volunteers that remain the key information providers, particularly when physical access becomes limited due to physical distancing measures. Based on this review, we advocate to further strengthen these approaches and recommends that they are implemented for the protection and promotion of health and well-being, including for health emergencies.

## Introduction

COVID-19 has affected nearly all countries in the world, by 28 September 2020 an over 33 million confirmed cases and a case fatality ratio of around 3% (1). The effect of COVID-19 pandemic is felt beyond health and incorporates a profound impact on all sectors within the society. The recent Ebola, Zika, and MERS-CoV outbreaks have demonstrated that the simplest path yet the most effective to organize and respond to health emergencies is to build trust and confidence in communities and services, understand community views and proactively share information and to work with communities to keep people safe (2).Thus, pandemic response interventions implemented by the government through community involvement is extremely needed (3–5). A study on lessons learned during Ebola outbreak highlights that to realize successful Community Engagement (CE), communities must be active participants in health response efforts and that communication platforms for CE be established ahead of a crisis (6). Both of these criteria are well-established in Oman.

Community participation is one of the main principles of primary health care (PHC), the strategy proposed in Almata in 1978 and adopted by member states and reaffirmed in 2018 by the Astana declaration (7, 8).

Community participation is defined as the process by which individuals and families assume responsibility for their own health and welfare and those of the community, and build their capacity to contribute to their and the community's development (9).

This review of community participation approaches in pandemic COVID19 in Oman is influenced by the two theoretical frameworks (10): “Continuum of community engagement approaches”; and the World Health Organization's (11) “Wheel of participation” conceptual framework.

Both frameworks emphasis the fact that community participation aims to empower local leaders, parents, families, groups, and the whole community. It involves planned actions to achieve, influence, and involve all relevant segments and sectors of society to realize a mutual goal. Thus, it goes beyond dialogue or interaction with selected groups to genuinely consult and empower all people, particularly the poor, deprived, and disadvantaged members of society.

The frameworks highlight the main feature of community participation in health is that individuals and community groups work together in partnership to take decisions to handle health-related issues and threats like pandemics and promote well-being to attain positive health outcomes.

The concept of community participation requires a highly participatory environment where community-based initiatives provide community structures and mechanisms to effectively enforce them.

A recent review reports extensive evidence that community participation, as multifaceted practices influenced by a range of social and cultural factors, has a positive effect on health, especially when corroborated by robust organizational and community processes (12). In Oman, CE is one of the pillars of the health strategies that are developed taken into account the social and economic determinants of health (13).

The primary two cases of COVID-19 were reported in Oman on the 24th of February 2020 and were related to travel to the Islamic Republic of Iran (14, 15). Since then, the number of confirmed cases has increased drastically to reach 97,450 cases with a mortality rate of 0.9% (*n* = 909) by the 28th of September 2020 (16).

In Oman, the Primary Health Care (PHC) is the basic building block for the health system and the designated facility where the patient's first contact with the healthcare system occurs and it incorporates a range of activities and where the community participation (CP) is a core component. Therefore, the existing CP mechanisms served as an important platform to engage communities in the COVID-19 pandemic preparedness and response (17). These mechanisms acted to liaise between the governmental bodies and the community and promoted the uptake of recommended protective behaviors, which reduced the transmission of infection at the local level.

This descriptive study aims to review and appraise the participatory community approaches in Oman during the pandemic response, to discuss the lessons learned and provide recommendations to strengthen community and inter-sectoral actions not only during emergencies but beyond that for better health and well-being.

## Community Participation for Health in Oman

In 1991, as a part of the PHC program, the MOH introduced the community participation approach (13) which has resulted in the establishment of a variety of Healthy Cities (HCs) and Healthy Villages (HVs), a network of Willayat (District) Health Committees (WHCs), and a group of community-based volunteers to implement a wide range of public health interventions, like involving in pH1N1 in 2009 and more recently in elimination of many of vaccine preventable diseases namely measles and rubella.

The common goal of these approaches is to make the political, social, and economic policies and plans of actions for all segments of the community that promote health and produces a positive impact on the environment and quality of life. These three platforms, Healthy Cities, Willayat Health Committees, and the Community Support Groups were the key approaches used for the COVID-19 response.

## Community Participation During COVID-19 Pandemic

Since COVID-19 was declared a worldwide pandemic by the World Health Organization (WHO) on the 11th of March 2020 (18), Oman adopted and implemented its preparedness and response plan for the pandemic (19, 20). The community involvement national response plans were activated and made available at all sub-national levels. In addition, the community participation approaches were activated to ensure proper networking and disseminating the necessary information at all levels.

**Figure 1:**
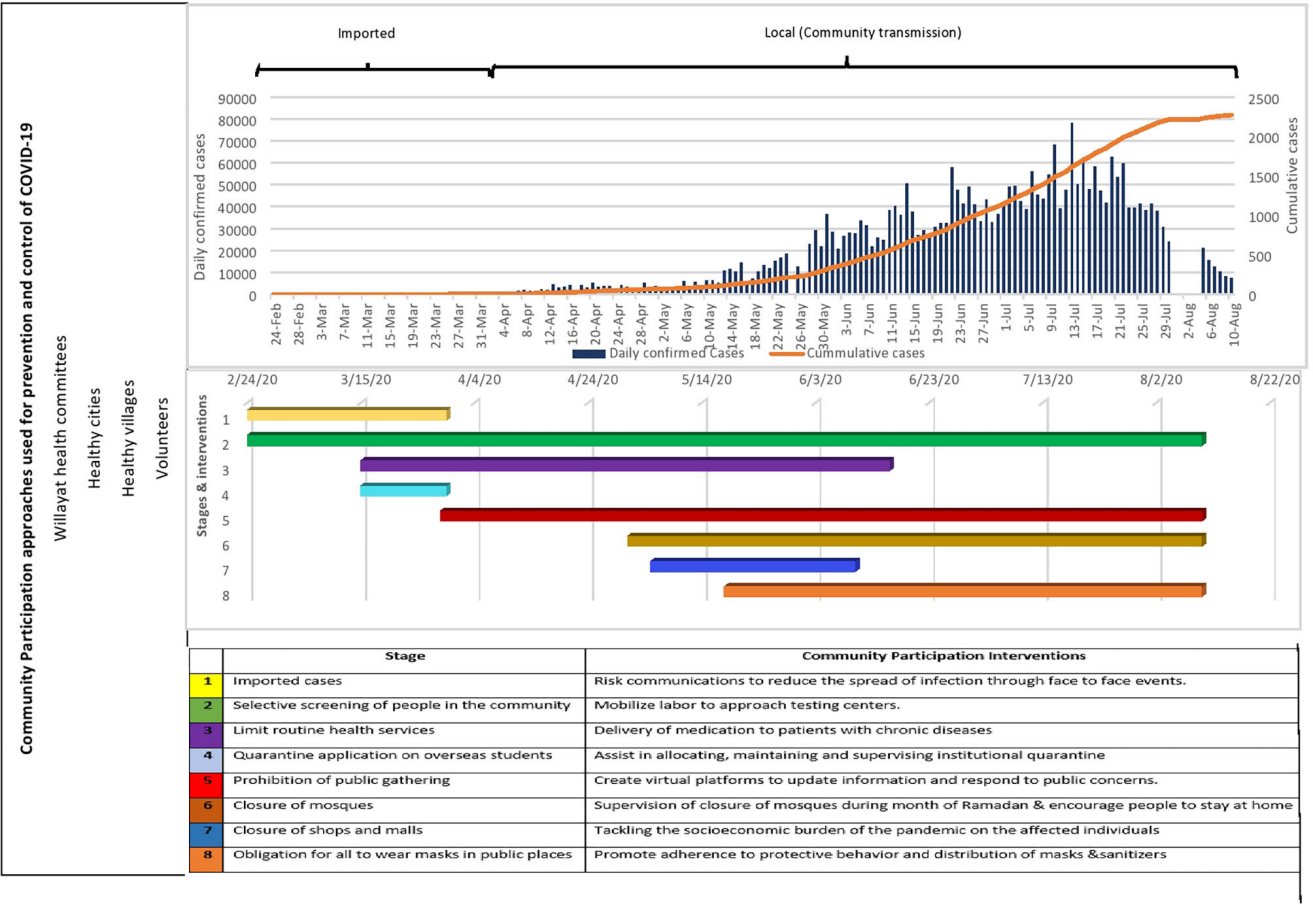
Community Participation approaches used for prevention and control of COVID-19, Willayat (District) healthy, Oman.

Health experts recognized the important role of communities to stop the spread of the diseases and manage the pandemic through non-pharmaceutical interventions until a vaccine and treatments are developed (21). Collective approaches to community participation can add value within the COVID-19 response by ensuring people are working within the right structure to deliver the best results and increase the effectiveness of interventions (22). Thus, the three community participation approaches were incorporated as a critical component in Oman's preparedness and response plan (Figure 1).

## The First Approach: Healthy Cities And Villages

The Healthy Cities and Healthy Villages Programme is concerned with improving the physical, social, spiritual, and economic dimensions of health. It addresses various social determinants of health using community resources, which enable people to mutually support each other in performing the different functions of life (23). Local communities are encouraged to collaborate with the various government and non-governmental agencies, allowing members of every community to play an active role in developing and improving their determinants of health. The community development committee, which is created at each city and village, is accountable for overseeing all program activities and taking decisions to ensure the betterment of the area and its population.

**Figure 2:**
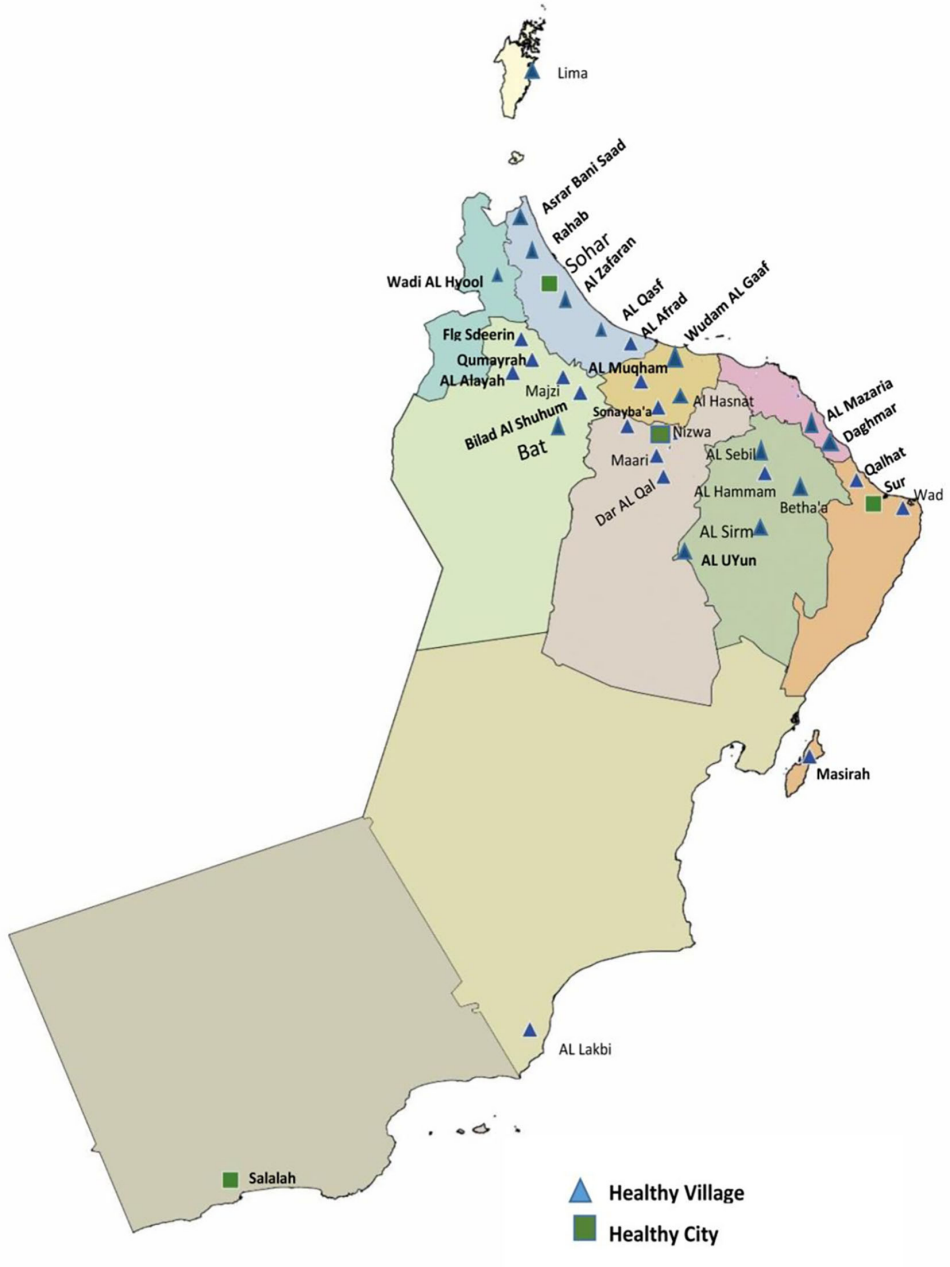
Distribution of healthy Cities and villages, Oman.

As of August 2020, there are 39 healthy villages and four HCs (24, 25). These sites use different steps of implementation, including community preparation, community organization, capacity building, situation analysis, and other activities (Figure 2). The HCs implementation package used was either developed by the WHO (24, 25), to guide the community in establishing a well-structured health city programme. The MOH provided guidance to this network of 43 sites to carry out collective and coordinated actions to mobilize the community in the COVID-19 pandemic response.

## Examples From SUR Healthy City

Cities are at the forefront of the crisis fostering the implementation of the national preventive measures; therefore, they need to develop plans which are tailored from the national preparedness and response plan (20). In Sur, certified by WHO as a healthy city in 2018 (25, 26), the city committee developed an interventional plan of action with key partners, including civil society, e.g., Omani Women Association, Scout, Sports Clubs, Community Support Groups, and Societies.

Each organization agreed to conduct a specific set of activities including supporting efforts to arrange, maintain, and supervise the institutional quarantine for COVID-19 affected individuals. The involvement of community leaders and social media influencers allowed the team to spread knowledge and disseminate different health education messages.

## Migrant Workers

In Sur city, as in any urban setting, the diversity of the population imposes challenges to the measures taken to deal with the pandemic. One of these challenges was the existence of a vulnerable population (27). The total population in Sur is 120,876 as per 2019 data, with 40% of them are expatriates (28). The majority of them are migrant workers, which are considered as a vulnerable group because many of them couldn't afford a proper, healthy quarantine place due to shared and overcrowded accommodation they have, in addition to the language barrier, which put them at a higher risk of infection and disease spread. A number of migrant workers do not have any legal documents; therefore, they usually do not seek medical advice even if they have symptoms.

The local committee in Sur was committed to allocate, supply and run the institutional quarantine (14 days) for this segment of the community including the patients and their contacts, in addition, identification of areas where they live and their environment. The local committee with the support from local community developed and disseminated information about the pandemic in the appropriate languages understood by migrant's workers. Moreover, efforts of the city were also directed to improve the socio-economic status of the individuals and families affected financially by the lockdown by providing them with the essential needs such as groceries, meals, masks, and sanitizers, in coordination with charity teams.

## Example From AL Buraimi Healthy City

### Ramadan Stay Home Initiatives

Al Buraimi HC responded to the pandemic by conducting a community-based initiative called “We are all responsible” aiming to heighten awareness of the whole community and scale down the spread of coronavirus and encouraging residents to remain at home during Ramadan (the holy fasting month). During this period, Muslims fast during daylight hours, congregate in prayers at night, and share meals with the community. But with the strict rules and physical distancing national regulations to limit the spread of COVID-19, many of Ramadan's rituals and traditions were either suspended or reduced by limiting the number of attendees. Consequently, many people felt that these restrictions would diminish the spirit and intent of Ramadan.

In response, the Buraimi initiative developed a communication campaign in the local community with ideas and ways on how to spend valuable and enjoyable times with their families and how to keep in touch with their distant family members despite the mobility restrictions. Examples include educational games for children and competing games for adults, communicating different preventive messages in the Arabic language which were disseminated through the social media platforms of the Directorate General of Health Services in Al Buraimi and through volunteer Whatssap^TM^ groups 2–3 times per week during Ramadan. This method was selected to deliver the correct information to a large segment of people in a safe manner and at the lowest cost. The initiative also included a link to register those wishing to donate blood through the blood bank at Al Buraimi Hospital, which positively impacted the number of blood donations.

## Example From Nizwa Healthy City Project

The project aimed to enhance community empowerment through communication and capacity building in coordination with civil society and academia. Nizwa Wilayat is located in Al Dakhliyah Governorate, with a total population of 131,108, around 32.5% of them are expatriates (28). During the pandemic, Nizwa Healthy city participated actively in building the capacity of community members on effective risk communications to reduce the spread of infection and support COVID-19 response efforts.

Updated and reliable information from the health workers allowed the local team in Nizwa to design, produce, and disseminate health education materials, which helped people understand what COVID-19 is and be aware of and comply with precautions measures. Three training workshops were conducted for 18 Rovers and volunteers on the key areas of developing health educational materials. Messages on nutrition, physical activity, smoking, mental health, and elderly care were also communicated through billboards and social media during the pandemic. This was done in collaboration with students from the University of Sharqiya and the Oman Anti-tobacco Society. To ensure that the community in Nizwa is not a passive actor, but rather has an active role in addressing and helping to resolve this health issue, a virtual training workshop was conducted, targeting local stakeholders, on the role of civil society organizations in emergency preparedness and management. Twelve members participated actively from the Omani women association, Oman cancer association, Alnoor association for the blind, sports club, elderly care association, and Nizwa Zakat and charity teams. Involving the local community and building their capacity in Nizwa were some of the key components in empowering people to reduce community transmission and enabled transparent decision making during the pandemic.

## The First Approach: Healthy Villages (HVS)

The existing HVs committees carried out numerous activities of risk communications to reduce the spread of infection through information on ways to protect the health of individuals and communities. Messages on prevention of COVID-19 were developed by the HVs and provided in multiple and accessible formats, including videos with linguistic communication for people with hearing difficulties. Educational materials were developed by the HVs and distributed throughout the village's social media channels, responding to the circulating rumors and false information about COVID-19.

In some villages, cars with microphones were accustomed to disseminate messages on preventive measure and asking people to remain at home. Other activities include supervising the closure of mosques and delivering medicines to people with chronic diseases in coordination with the health institution, analyzing the socio-economic situation of the affected families, and providing them with the basic needs of food, drinks, and other household supplies. Following the closure of barbershops, some local committees in the villages distributed shaving tools for each family in the village and provided them with training on how to use them.

## The Second Approach: Willayat Health Committees (WHCs)

WHCs were established by MOH in 1999 to engage the community and other government sectors in identifying social and healthy lifestyle challenges and to suggest solutions (29). There are 61 WHCs in Oman, one in each willayat, each headed by the Wali (head of the district) and include members from government sectors, civil societies, and, therefore the community. Each committee coordinates with the MOH, and related sectors and the community to address the social determinants of health within the Willayat. People from different sectors and various segments of society are engaged in dialogue and negotiation for collective and collaborative actions. Additionally, governmental departments, organizations, stakeholders, opinion-makers, and political leadership are organized into partnerships and through collaborative actions toward the goal of community development. WHCs are accustomed to mobilize communities and promote active participation in assessing their needs and solving their problems through community-based projects (30).

During the COVID-19 pandemic, the system of WHCs acted as a platform for intersectoral collaboration for health promotion at Willayat (district) level aiming to involve grassroots community leaders in the preparations and implementations of the outbreak response. These committees engaged in implementing the recommendations of the Supreme COVID-19 Committee at the local level.

In all districts, WHCs played a vital role in the efforts to confront the pandemic. Following stakeholder mapping, communication plans were formulated by the members of the WHC, and volunteers were trained on ways to raise awareness about the pandemic in the community, especially in areas where the health institutions do not have a health education cadre (31). Furthermore, WHCs coordinated different sectoral and community activities and provided the political will and support, particularly, to tackle the socio-economic burden of the pandemic on affected families and vulnerable segments of the community.

## The Third Approach: Volunteers

Community Support Groups (CSGs) are groups of 4,000 women and men volunteers who work as links between the community and the health system to promote individual health and community health (32).

CSGs were established in 1992 to promote breastfeeding; since then, their mandate has expanded and they have become an integral component of the primary health care network in the country to raise awareness of health-related issues within the community.

The volunteers work in coordination and under the supervision of the health center in their catchment area or through the WHCs or the local development committees in areas implementing HV or HC programs. Therefore, the MOH took special attention and interest in these volunteering groups (~4,000) through regular support, training, motivation, instruction, and supervision (13).

## The Role of Volunteers During the Pandemic

CSG volunteers had 3 critical roles: (a) Health educators; disseminating knowledge on preventive measures (cough etiquette, hand washing, and physical distancing) and quarantine procedures, and the importance of adherence to the restriction of movements. They also assist in the response to public perceptions, worries, concerns, rumors, and mixed and confusing messages that can impact operational communications; (b) Data collectors; maintaining data records on individuals in the institutional quarantine, contact tracing, conducting situation analysis about affected families and identifying specific risks for various groups, and; (c) Social mobilizers; help with mobilizing the expatriate labor workers to approach the testing centers, and to seek medical advice when symptomatic, and conducting fundraising and blood donation campaigns. This was done in coordination with embassies, clubs, and group leaders of different nationalities.

At the early stage (imported cases reported) of the pandemic face to face meetings, events and workshops were carried out by the volunteers, but later digital channels were used for communications and community engagement.

## Discussion

Although, it is difficult to ascertain the direct link between community-based interventions and health outcomes during the pandemic, however, their importance and contribution could be discussed from the lessons learned using community participation approaches in Oman to combat several other health problems in the country since 1991: First, community organizations provide a good understanding of the community and facilitate the involvement and collaboration with various segments of the community. Second, the bottom-up approach, through community participation, permits people to identify their needs and take appropriate actions to fulfill them. This consecutively ensures ownership and maintain sustainability. Third, well-planned media and mass communication methods enhance the ability to diffuse information successfully through different community networks. Fourth, these approaches ensure efficient mobilization of resources within the community, including financial, in-kind materials, and manpower. Fifth, reliable monitoring and evaluation systems are vital components for the demonstration of change.

Similar experiences have been documented in other countries. For example, a rapid evidence review was conducted earlier this year to identify how community engagement is used for infectious disease prevention and control during epidemics of Ebola, Zika, SARS, MERS, and pH1N1 since 2000. The review identified 37 initiatives where community engagement was employed for different stages of risk reduction including in planning, gaining community entry and strengthening confidence, risk communication, and surveillance and tracing, among others. The review encourages countries to assess existing community engagement structures and to use them to support COVID-19 control measures.

The role of community engagement in pandemic response to COVID-19 in Oman was evident and has benefited from the long experience in the country employing community participation as highlighted in the above lesson learned. The current review identified the potential role community engagement played in containing the spread of the COVID-19 pandemic in Oman.

The three approaches of community participation in Oman provide different ways of engaging community members in protecting their health during the pandemic and come with distinctive strengths. One major strength is the linkages between the three approaches, especially through the use of the volunteers of Community Support Groups (CSGs) (33). Another strength is the reliance of these community approaches on a unified national system for guidance/advice/materials, especially for the communication messages/campaigns. This is in addition to the innovative ways that individual communities take in addressing their needs, for example, the use of cars mounted with microphones or digital channels for raising awareness.

## Conclusion

Based on the findings of this review, we recommend to strengthen the existing community participation mechanisms, to establish new approaches and partnerships, and to build the capacities of local stakeholders in supporting communities to respond to different health challenges and threats.

## Data Availability Statement

The original contributions generated for the study are included in the article/supplementary material, further inquiries can be directed to the corresponding author/s.

## Author Contributions

All authors listed have made a substantial, direct and intellectual contribution to the work, and approved it for publication.

## Conflict of Interest

The authors declare that the research was conducted in the absence of any commercial or financial relationships that could be construed as a potential conflict of interest.
